# Rapid-deployment aortic valve replacement in high-risk patients: A case-control study

**DOI:** 10.34172/jcvtr.2021.10

**Published:** 2021-01-30

**Authors:** Adama Sawadogo, An Vinh Bui-Duc, Nicolas D'Ostrevy, Lionel Camilleri, Kasra Azarnoush

**Affiliations:** ^1^Department of Cardiovascular, University Hospital of Clermont-Ferrand, France; ^2^Department of Cardiovascular and Thoracic Surgery, University Hospital of Tengandogo, Burkina Faso; ^3^Department of Cardiac Surgery, E Hospital of Hue, Vietnam

**Keywords:** Aortic Valve Replacement, Rapid Deployment Aortic Valve, Calcified Aortic Stenosis, EuroSCORE II

## Abstract

***Introduction:*** Aortic valve stenosis is the most frequent cardiac valve pathology in the western world. In high-risk patients, conventional aortic valve replacement (C-AVR) carries high rates of morbidity and mortality. In the last few years, rapid-deployment valves (RDV) have been developed to reduce the surgical risks. In this work, we aimed to compare the mid-term outcomes of rapid-deployment AVR (RD-AVR) with those of the C-AVR in high-risk patients.

***Methods:*** This retrospective case-control study identified 23 high-risk patients who underwent RD-AVR between 12/2015 to 01/2018. The study group was compared with a control group of 46 patients who were retrospectively selected from a database of 687 C-AVR patients from 2016 to 2017 which matched with the study group for age and Euro SCORE II.

***Results:*** RD-AVR group presented more cardiovascular risk factors. Euro SCORE II was higher in the RD-AVR group (*P* =0.06). In the RD-AVR group, we observed significantly higher mean prosthetic size (*P* <0.001). In-hospital mortality was zero in RD-AVR group versus 2 deaths in C-AVR group. Hospital stay was longer in the RD-AVR group with statistical significance (*P* =0.03). In the group AVR with associated cardiac procedures, while comparing subgroups RD-AVR versus C-AVR, early mean gradient was lower in the first cited (*P* =0.02). The overall mean follow-up was 10.9 ± 4.3 months.

***Conclusion:*** The RD-AVR technique is reliable and lead to positive outcomes. This procedure provides a much larger size with certainly better flow through the aortic root. It is an alternative to C-AVR in patients recognized to be surgically fragile.

## Introduction

Aortic valve stenosis is the most frequent cardiac valve pathology in the western world and aortic valve replacement (AVR) is the standard treatment.^1^ Recently, in this era of an aging population, the presentation of older and sicker patients with heavily calcified valves, root calcification and diffuse atherosclerosis, diabetes and redo is increasing.^2^ As standard AVR is associated with high rates of morbidity in these patients, in the last few years, rapid-deployment aortic valves (RDV) have been developed to facilitate surgical methods, reduce aortic cross-clamping and cardiopulmonary bypass (CPB) time and curtail the risk of mortality and morbidity, and to maintain satisfactory hemodynamic outcomes and low periprosthetic leak rates.^3,4^ Several studies confirmed how RDV has been widely used even in off-label indications such as with concomitant mitral valve surgery,^5^ surgery for endocarditis,^6^ isolated aortic regurgitation^7^ and may also be an alternative for high-risk patients.^8^

In this study, we compared the mid-term outcomes of rapid-deployment AVR (RD-AVR) with those of the conventional procedure in high-risk surgical patients who underwent isolated AVR or with other cardiac procedures.

## Materials and Methods

### Patients

Between December 2015 to January 2018, 23 high-risk surgical patients underwent RD-AVR according to the following criteria: age ≥ 64 years old, multiple procedures on extremely fragile patients at the University Hospital of Clermont-Ferrand, France. In some patients, the decision to use the RD-AVR was made peroperatively because of anatomical and pathological findings like huge valve calcification and small aortic root that were making conventional AVR (C-AVR) impossible or risky to perform. The study group was compared with a control group of 46 patients who were retrospectively selected from a database of 687 C-AVR patients from 2016 to 2017 which matched with the study group for age and EuroSCORE II (European System for Cardiac Operative Risk Evaluation II). Table 1 shows the preoperative data of all included patients. Patient age ranged from 64 to 84 years. In the RD-AVR group, we implanted an Intuity Elite valve (Edwards Lifesciences LLC, USA) in 10 patients and a Perceval valve (LivaNova PLC, UK) in 13 patients. In the C-AVR group, we placed a Perimount Magna Ease (Edwards Lifesciences LLC) in 24 patients, a Trifecta valve (St. Jude Medical, USA) in 20 patients and a Crown PRT valve (Sorin Group, Canada) in 2 patients. Follow-up: the patients were followed up and a telephone call was given to the cardiologists in both groups to collect the latest information. The questions included postoperative complications and latest echocardiography findings. The data were collected according to the EACTS/STS guideline for reporting cardiac events after valve surgery ^9^.

**Table 1:** Patient’s preoperative characteristics

**Patients**	**Total** **(n = 69)**	**RD-AVR group** **(n = 23)**	**C-AVR group** **(n = 46)**	***P*** ** value**
Age, mean ± SD, y	75.7 ± 5.2	75.8 ± 5.7	75.6 ± 5.0	0.88
Male, n (%)	62 (89.9)	21 (91.3)	41 (89.1)	1.00
Body mass index, mean ± SD, kg/m²	28.7 ± 4.6	28.8 ± 5.0	28.7 ± 4.4	0.91
NYHA class, n (%)				1.00
I - II	36 (52.2)	12 (52.2)	24 (52.2)	
III - IV	33 (47.8)	11 (47.8)	22 (47.8)	
Unstable angina Class IV, n (%)	4 (5.8)	3 (13.0)	1 (2.2)	0.10
Critical preoperative state, n (%)	3 (4.4)	1 (4.4)	2 (4.4)	1.00
Urgency, n (%)	13 (18.8)	6 (26.1)	7 (15.2)	0.33
Previous cardiac surgery, n (%)	4 (5.8)	3 (13.0)	1 (2.2)	0.10
Recent myocardial infarction, n (%)	2 (2.9)	2 (8.7)	0 (0)	0.11
Arterial hypertension, n (%)	54 (78.3)	20 (87.0)	34 (73.9)	0.22
Dyslipidemia, n (%)	52 (75.4)	19 (82.6)	33 (71.7)	0.32
Smoking, n (%)	22 (31.9)	6 (26.1)	16 (34.8)	0.47
Extracardiac arteriopathy, n (%)	10 (14.5)	8 (34.8)	2 (4.4)	0.002
Chronic lung disease, n (%)	5 (7.3)	2 (8.7)	3 (6.5)	1.00
Diabetes, n (%)	34 (49.3)	12 (52.2)	22 (47.8)	0.73
Endocarditis active, n (%)	4 (5.8)	2 (8.7)	2 (4.4)	0.60
Renal function, n (%)				0.11
Normal	35 (50.7)	11 (47.8)	24 (52.2)	
Moderately impaired	27 (39.1)	7 (30.4)	20 (43.5)	
Severely impaired	5 (7.3)	4 (17.4)	1 (2.2)	
On dialysis	3 (2.9)	1 (4.4)	1 (2.2)	
Echocardiogram				
LVEF, n (%)				0.76
Normal (>50%)	55 (79.7)	19 (82.6)	36 (78.3)	
Mild/moderate impairment (30 – 50%)	14 (20.3)	4 (17.4)	10 (21.7)	
Severe impairment (<30%)	0 (0)	0 (0)	0 (0)	
Pulmonary hypertension, n (%)				0.41
Normal (<31 mm Hg)	44 (63.8)	15 (65.2)	29 (63.0)	
Moderate (31 – 55 mm Hg)	22 (31.9)	6 (26.1)	16 (34.8)	
Severe (>55 mm Hg)	3 (4.4)	2 (8.7)	1 (2.2)	
Mean aortic gradient (mm Hg), mean ± SD	39.8 ± 14.8	38.4 ± 15.2	40.5 ± 14.6	0.59
Aortic regurgitation ≥ grade II, n (%)	13 (18.8)	3 (13.0)	10 (21.7)	0.52
EOA (cm^2^), mean ± SD	0.8 ± 0.2	0.8 ± 0.3	0.8 ± 0.2	0.83
EUROSCORE II, median [IQR]	2.5 [1.8; 3.6]	2.9 [2.2; 4.0]	2.2 [1.6; 3.5]	0.06

Abbreviations: SD, standard deviation; NYHA, New York Heart Association; LVEF, left ventricular ejection fraction; EOA, effective orifice area; EUROSCORE II, European System for Cardiac Operative Risk Evaluation II; IQR, interquartile range; RD-AVR, rapid deployment - aortic valve deplacement ;C-AVR, conventional - aortic valve deplacement

### Operative technique

The C-AVR procedure is performed as fully described in our previous work ^10^ : native aortic valve resection, sizing, and valve implantation by inverted and interrupted pledgeted suture with Cardioxyl® 2/0 (Peters Surgical, France). For the RD-AVR procedure, the aortic orifice was measured with the original sizer of the bioprosthesis. Three 4-0 polypropylene guiding sutures were passed at the nadir of the aortic annulus. The three guiding sutures were passed through the three holes arising from the annular ring of the prosthesis. Once the delivery system is in position, the valve is deployed by turning the release screw and placing the valve in place. Then the delivery system is removed. The field was rinsed with warm saline, and the prosthesis was dilated according to the recommendations.^11^ After closing of the aortotomy, transesophageal echocardiography was performed to assess the correct implantation of the prosthesis and the presence of any leakage.

## Statistical analysis

Statistical analysis was performed using Stata software (version 13; StataCorp, College Station, Texas, USA) and R 3.3.1 (http://cran.r-project.org). All tests were two-sided, with a Type I error set at 0.05. Patients were retrospectively matched (2 patients with the conventional valve for 1 patient with RDV) according to age, gender, EuroSCORE II and associated gesture. This was made by the method of the nearest neighbor. Categorical parameters were expressed as frequencies and associated percentages and continuous data as the mean ± standard deviation or as median [interquartile range], according to the statistical distribution. Categorical variables were compared between independent groups (RD-AVR or C-AVR) using the chi-square test or the Fisher exact test. Quantitative data were compared between groups with the Student t test or with the Mann-Whitney test, as appropriate. The Gaussian distribution was verified by the Shapiro-Wilk test and homoscedasticity by the Fisher-Snedecor test. Adjustments for age, associated gesture or EuroSCORE II were also considered in a multivariate point of view (linear or logistic regression, according to the nature of the dependent variable). Interactions between these factors and the two groups of patients (RD-AVR or C-AVR) were also studied. Subgroup analyses were then performed.

## Results

Table 1 shows the overall patient’s preoperative data. The group of RD-AVR presented more cardiovascular risk factors, including unstable angina, previous cardiac surgery, recent myocardial infarction, hypertension, dyslipidemia, lung diseases, diabetes, endocarditis, renal impairment, and peripheral arterial disease. All these criteria were not statistically significant except for the latter (*P* = 0.002). EuroSCORE II was higher in the RD-AVR group: 2.9 [2.2; 4.0] vs. 2.2 [1.6; 3.5] respectively, for a median of the RD-AVR and C-AVR (*P* = 0.06).

Table 2 shows the intraoperative results.In the RD-AVR group, we observed significantly higher mean prosthetic size: 24.6 ± 2.2 mm vs. 22.6 ± 2.1 mm (*P* < 0.001). The most frequent associated procedure was CABG in both groups. There was no difference in term of CPB and cross-clamp time in both groups.

**Table 2:** Intraoperative data

	**Total** **(n = 69)**	**RD-AVR group** **(n = 23)**	**C-AVR group** **(n = 46)**	***P *** **value**
Type of procedure, n (%)				0.96
Isolated AVR	17 (24.6)	5 (21.7)	12 (26.1)	
AVR + other procedures				
Ascending aortic surgery	1 (1.5)	0 (0)	1 (2.2)	
MVR	2 (2.9)	1 (4.4)	1 (2.2)	
MVR + Tricuspid repair	3 (4.4)	1 (4.4)	2 (4.4)	
Tricuspid repair	2 (2.9)	0 (0)	2 (4.4)	
CABG	39 (56.5)	15 (65.2)	24 (52.2)	
CABG + ascending aortic surgery	2 (2.9)	1 (4.4)	1 (2.2)	
CABG + MVR	2 (2.9)	0 (0)	2 (4.4)	
CABG + Tricuspid repair	1 (1.5)	0 (0)	1 (2.2)	
Prosthetic size, n (%), mm				0.004
19 mm	7 (10.1)	1 (4.4)	6 (13.0)	
21 mm	13 (18.8)	2 (8.7)	11 (23.9)	
23 mm	21 (30.4)	4 (17.4)	17 (37.0)	
25 mm	21 (30.4)	10 (43.5)	11 (23.9)	
27 mm	7 (10.1)	6 (26.1)	1 (2.2)	
Mean prosthetic size, mean ± SD, mm	23.2 ± 2.3	24.6 ± 2.2	22.6 ± 2.1	<0.001
CPB time, mean ± SD, min	121.9 ±42.7	121.7 ± 30.3	122.0 ± 48.0	0.67
Cross-clamp time, mean ± SD, min	95.9 ±35.0	91.0 ± 27.9	98.4 ± 38.2	0.57

Abbreviations: AVR, aortic valve replacement; MVR, mitral valve replacement; CABG, coronary artery bypass grafting; CPB, cardiopulmonary bypass; SD, standard deviations;RD-AVR, rapid deployment - aortic valve deplacement ;C-AVR, conventional - aortic valve deplacement

In-hospital mortality rate was low (2.9%) and concerned 2 patients in the conventional group (*P* = 0.55). There was no death in the RD-AVR group. Both patients died in the ICU, one for multiorgan failure on the same day of operation and the other one suffered from sudden cardiac arrest on postoperative day 9 despite cardiopulmonary resuscitation and emergency resternotomy. Table 3 shows the core of postoperative outcomes. ICU and hospital stay were longer in the RD-AVR group with statistical significance (*P* = 0.02, *P* = 0.03). On discharge echography, there was some mild/trivial periprosthetic leak (17.4%) in the RD-AVR group, but not requiring reoperation (*P* = 0.08). The mean gradient was slightly lower in the same group (*P* = 0.14). Regarding the subgroup AVR with associated cardiac procedures, the early postoperative mean gradient was 8.0 ± 3.2 mm Hg with the RD-AVR group and 11.3 ± 4.9 mm Hg C-AVR group (*P* = 0.02). All patients were free from aortic regurgitation > grade 2.

**Table 3:** Postoperative outcomes and discharge echography

	**Total** **(n = 69)**	**RD-AVR group** **(n = 23)**	**C-AVR group** **(n = 46)**	***P*** ** value**
In-hospital mortality, n (%)	2 (2.9)	0 (0)	2 (4.4)	0.55
Postoperative complications				
Reintervention	7 (10.1)	2 (8.7)	5 (10.9)	1.00
Bleeding, tamponade	4 (5.8)	2 (8.7)	2 (4.4)	
Cardiac arrest	2 (2.9)	0 (0)	2 (4.4)	
Veno-venous ECMO support	1 (1.5)	0 (0)	1 (2.2)	
Heart rythm				
Atrial fibrillation or flutter	20 (29.4)	5 (21.7)	15 (33.3)	0.32
Permanent pacemaker	10 (14.7)	6 (26.1)	4 (8.9)	0.08
Stroke	1 (1.5)	0 (0)	1 (2.2)	1.00
Dialysis	1 (1.5)	0 (0)	1 (2.2)	1.00
ICU stay, median [IQR], days	3 [2; 5]	4 [2; 5]	2 [1; 4]	0.02
Hospital stays, median [IQR], days	14.5 [11; 21]	17 [13; 25]	12 [10; 18]	0.03
Discharge echography				
LVEF, mean ± SD, (%)	60.3 ± 9.3	58.7 ± 9.1	61.1 ± 9.4	0.33
Early Prosthetic leakage				0.08
No leak	58 (89.2)	19 (82.6)	39 (92.9)	
Periprosthetic leak	5 (7.7)	4 (17.4)	1 (2.4)	
Intraprosthetic leak	1 (1.5)	0 (0)	1 (2.4)	
Both	1 (1.5)	0 (0)	1 (2.4)	
Mean aortic gradient, mean ± SD, mm Hg	10.4 ± 4.5	9.3 ± 4.5	11.0 ± 4.5	0.14

Abbreviations: ECMO, extracorporeal membrane oxygenation; ICU, intensive care Unit; IQR, interquartile range; LVEF, left ventricular ejection fraction; IQR, interquartile range; SD, standard deviations; RD-AVR, rapid deployment - aortic valve deplacement ;C-AVR, conventional - aortic valve deplacement

Follow-up was completed in 100% of the patients. During the mean follow-up of 10.9 ± 4.3 months, there were no deaths, LVEF remained normal and mean gradient lower in the RD-AVR group (*P* = 0.32, *P* = 0.50).

## Discussion

The key finding of our study is the relatively good postoperative and mid-term outcomes regardless of the preoperative risk level. Like other series of RD-AVR, the majority of our patients were in the third age.^5,7^ Peripheral arterial disease, more found in the RD-AVR group represented the main cardiovascular risk factor that was statistically significant. Indeed, aortic root calcification is one of the relative contraindications of C-AVR and many studies have demonstrated the association between arterial calcification and cardiovascular risk.^12^ In our study, preoperative data showed the predominant risk factors in the RD-AVR group; these patients were probably subject to diffuse arterial calcification.

Although EuroSCORE II confirmed the higher score in the RD-AVR group compared to the control group, the postoperative course went well as no death was noticed in this risky group (*P* = 0.06). C-AVR in patients with a small aortic annulus may result in the prosthesis mismatch which is more common in smaller patients, obese and elderly women with multiple comorbidities.^13^ This procedure provides a much larger size with certainly better flow through the aortic root as the mean prosthetic size was greater in this group.^4^

Intraoperatively, CPB and cross-clamp time were similar. In fact, operative time in the group of the high-risk patient was supposed to be longer if the procedure was C-AVR. As previously demonstrated by Flameng et alRD-AVR can reduce cross-clamp time by 18 ± 6 minutes in case of isolated AVR.^14^ Standing on our early results, we can state that RD-AVR is an efficient technique with good results, at least equivalent to C-AVR. It is easy to perform and can be an alternative to C-AVR in some particular situations such as redo, endocarditis and heavily calcified aortic valve. Although we overcame to reduce intraoperative time, ICU and hospital stay remained longer in the RD-AVR group. The reason is the patients of this group were presenting more comorbidity that is subject to require lasting management.

In the discharge echography, we could see that RD-AVR provided an improved cardiac output as demonstrated by a lower mean gradient in this group. This result remained good during the follow-up. A subgroup analysis of patients underwent AVR with concomitant procedures revealed a significantly lower mean gradient in the RD-AVR group, no more deterioration of the mean gradient was found. The new prosthesis was stable in the mid-term in both groups. Interestingly, there were no more valve-related events or other major complications in the high-risk group.

Our study presents some limitations. The term “high-risk” patient is controversial, neither EuroSCORE II nor STS scores alone are sufficient to define this specific status. The procedures were performed by different surgeons in our center. Like any other retrospective study, the data were collected after the procedures have been done. The size of the study population, especially the RD-AVR group was small. Another issue was the fact that echocardiogram reports were performed by different cardiologists and some parameters that are linked to personal interpretation may be different from one to another.

## Conclusion

The RD-AVR technique is reliable and lead to positive outcome at least equivalent to C-AVR. This procedure provides a much larger size with certainly better flow through the aortic root. It is an alternative to C-AVR in patients recognized preoperatively to be surgically fragile or peroperatively.

## Acknowledgements

The authors are thankful to Mrs Valerie Batel for her valuable help during this work.

## Competing interest

The authors have no conflicts of interest to declare.

## Ethical approval

The study was approved by institutional ethics committee of the University Hospital of Clermont-Ferrand (ETSH1200331A) and informed consent was taken from all the patients.

## Funding

None.
